# Seed Biopriming with Microbial Inoculant Triggers Local and Systemic Defense Responses against *Rhizoctonia solani* Causing Banded Leaf and Sheath Blight in Maize (*Zea mays* L.)

**DOI:** 10.3390/ijerph17041396

**Published:** 2020-02-21

**Authors:** Shailendra Singh, Udai B. Singh, Deepti Malviya, Surinder Paul, Pramod Kumar Sahu, Mala Trivedi, Diby Paul, Anil Kumar Saxena

**Affiliations:** 1 Plant-Microbe Interaction and Rhizosphere Biology Lab, ICAR-National Bureau of Agriculturally Important Microorganisms, Kushmaur, Maunath Bhanjan 275103, India; singh.shailendra512@gmail.com (S.S.); udaiars.nbaim@gmail.com (U.B.S.); deeptimalviya77@gmail.com (D.M.); surinderpaulsandhu@gmail.com (S.P.); pramod15589@gmail.com (P.K.S.); saxena461@yahoo.com (A.K.S.); 2 Amity Institute of Biotechnology, Amity University Uttar Pradesh, Lucknow 227105, India; 3 Pilgram Marpeck School of Science, Technology, Engineering and Mathematics, Truett McConnel University, 100 Alumni Dr., Cleveland, GA 30528, USA; dpaul@truett.edu

**Keywords:** seed bio-priming, microbial inoculant, anti-oxidative defense enzymes, *Rhizoctonia solani*, banded leaf and sheath blight, maize (*Zea mays* L.)

## Abstract

Plant growth promoting rhizobacteria *Pseudomonas aeruginosa* strain MF-30 isolated from maize rhizosphere was characterized for several plant growth stimulating attributes. The strain MF-30 was also evaluated for antifungal properties against *Rhizoctonia solani* causing banded leaf and sheath blight in maize (*Zea mays* L.) under in vitro conditions and was found to have higher mycelial growth suppression in the culture suspension (67.41%) followed by volatile organic compounds (62.66%) and crude extract (51.20%) in a dual plate assay. The endophytic and epiphytic colonization ability was tested using Green Fluorescent Protein (GFP)-tagging. Visualization through confocal scanning laser microscope clearly indicated that strain MF-30 colonizes the root and foliar parts of the plants. Further, the effects of seed bio-priming with *P. aeruginosa* MF-30 was evaluated in the induction and bioaccumulation of defense-related biomolecules, enzymes, natural antioxidants, and other changes in maize under pot trial. This not only provided protection from *R. solani* but also ensured growth promotion under pathogenic stress conditions in maize. The maximum concentration of hydrogen peroxide (H_2_O_2_) was reported in the root and shoot of the plants treated with *R. solani* alone (8.47 and 17.50 mmol mg^−1^ protein, respectively) compared to bioagent, *P. aeruginosa* MF-30 bio-primed plants (3.49 and 7.50 mmol mg^−1^ protein, respectively). Effects on total soluble sugar content, total protein, and total proline were also found to enhanced significantly due to inoculation of *P. aeruginosa* MF-30. The activities of anti-oxidative defense enzymes phenylalanine ammonia lyase (PAL), ascorbate peroxidase, peroxidase, superoxide dismutase, and catalase increased significantly in the plants bio-primed with *P. aeruginosa* MF-30 and subsequent foliar spray of culture suspension of MF-30 compared to pathogen alone inoculated plants. qRT-PCR analysis revealed that seed bio-priming and foliar application of *P. aeruginosa* MF-30 significantly increased the expression of PR-1 and PR-10 genes with the simultaneous decrease in the disease severity and lesion length in the maize plants under pathogenic stress conditions. A significant enhancement of shoot and root biomass was recorded in MF-30 bio-primed plants as compared to untreated control (*p* < 0.05). Significant increase in plant growth and antioxidant content, as well as decreased disease severity in the *P. aeruginosa* MF-30 bio-primed plants, suggested the possibility of an eco-friendly and economical means of achieving antioxidants-rich, healthier maize plants.

## 1. Introduction

Maize is one of the important cereal crops grown throughout the world for food, feed, and fuel. Maize production is often affected by the biotic and abiotic stresses devastatingly affecting the yield and quality of the produce and interferes in achieving the potential yield of a cultivar [1,2,3,4,5]. Hence, there is a need of new maize varieties with a strong genetic base for disease resistance [6,7,8,9] and high yield [1,2] and quality produce [1,2]. Further, in order to reduce the losses caused by insect pest and diseases, while simultaneously increasing the production, various research approaches on management strategies are being developed and carried out [1,2,6]. Among various diseases, banded leaf and sheath blight (BLSB) caused by *Rhizoctonia solani* (formally known as *Rhizoctonia sasakii*) is considered as one of the emerging and severe pathogens limiting the crop production under a changing climatic scenario [10,11,12]. The pathogen *R. solani* is a necrotroph which produces phytotoxins during the infection process causing necrotic spot on the leaf, sheath, and stem, preferably [1,2,6]. Under conducive environmental conditions, it colonizes almost all aerial parts of the plants including cobs and tassels. It has also been reported that heavily infected cobs did not produce grains and sometimes resting spores (sclerotia) are formed inside the cobs [1,10,11,12]. BLSB is favored by a warm and humid climate when the relative humidity ranges between 75% and 90%, as is the prevailing case during monsoon season in India [1,2,12]. For the management of BLSB, the major focus is on host plant resistance where maize germplasm is evaluated against *R. solani* at different agro-climatic zones to identify resistant sources [6,13,14,15,16]. To date, a small number of germplasm/donor parents have been identified as having resistance gene(s)/quantitative trait loci (QTLs), but the breakdown of resistance in due course of time is of great concern and poses a major challenge for plant breeders [17,18,19,20,21].

Now, the focus has been set on developing microbe-based strategies for the management of devastating pathogens along with resistant cultivars [1,2,22,23,24,25]. Application of antagonistic endophytic microorganisms is another important aspect of crop protection [26,27,28,29]. The endophyte induces beneficial effects in the plants via a number of complex biochemical signaling processes [27,28,29]. In general, the mechanisms employed by endophytes include the production of plant growth regulators, such as auxins, cytokinins, abscisic acid, and gibberellins, as well as secretion of effector molecules and secondary metabolites through modulation of various pathways/cascades [30,31,32,33]. These biomolecules and their interactions elicit the beneficial interaction of endophytes with plants and provide resistance to biotic stresses [33]. The beneficial plant–endophyte associations yielded in more efficient nutrient uptake, improved growth and development, and enhanced resistance to biotic stresses by eliciting plant defense systems [28,29,33].

Endophytic microorganisms can also act as biocontrol agents that help plants to defend themselves against pathogens attack [33]. These properties make endophytes attractive biocontrol agents for natural organic farming. These endophytes employ several mechanisms to kill/suppress the pathogen growth and colonization by direct parasitism, antibiosis, and/or competition for resources on the infection sites [28,29]. The indirect mechanisms involve the elicitation of systemic resistance responses in the host [33]. Induced systemic resistance (ISR) is generally elicited upon endophytic colonization in the roots system and protects plants on subsequent invasion caused by the pathogen [34,35,36,37]. The majority of plants primed with endophytes elicited ISR via modulation of defense networks/pathways including phenylpropanoid for resistance against upcoming pathogen attacks [38,39,40,41]. Endophytes modulate the production and synthesis of some important phytohormones, i.e., salicylic acid, jasmonic acid (JA), and ethylene (ET), which play a key role in initiating a stronger and faster resistance response following pathogen attack [33]. Further, some phytohormones, such as auxins, cytokinins, abscisic acid (ABA), gibberellins (GAs), and ET, also play a role in the establishing the relationship between the host plants and the endophytes [33].

Hormonal cross-talk is implicated not only in plant defense responses and endophyte interactions but, in general, it also regulates plant growth and development. Under the presence of compatible microbes in the plant system, fine-tuning takes place in the hormonal balance that leads to better plant growth, development, and reproduction even under pathogenic stresses [38,39,42,43]. Among rhizospheric and endophytic plant growth-promoting bacteria, *Pseudomonas aeruginosa* is the most studied and successful plant symbiont conferring resistance against a wider range of plant pathogens by direct antagonism or by triggering systemic resistance [44,45]. *P. aeruginosa* has adapted to a wide ecosystem and plays a major role in plant health [46,47,48]. It colonizes rhizospheric soil, roots, and in some cases aerial parts of the plant. It can even grow as endophytes [45,47,48,49]. *P. aeruginosa* is an excellent endo-symbiont and has myco-parasitic ability, protecting plants directly from pathogen attack, and it is well known for its ability to stimulate plant growth and development [48,49,50]. However, the role of endophytic *P. aeruginosa* in the *R. solani*-maize pathosystem is untouched and needs in-depth study. Besides the nature of crops and environmental conditions, the methods of inoculation influence the survival, multiplication, and colonization potential of microbial inoculants applied [51]. Among different methods of inoculation, seed treatments, seedlings treatments, foliar application, and soil treatments were widely used and found effective [52,53,54,55]. Seed bio-priming is a pre-sowing treatment which leads to a physiological state that enables microbial inoculants to establish a close contact with the seed [56,57]. The primed seeds exhibited faster and more synchronized germination. The young seedlings raised from bio-primed seeds are often more vigorous and resistant to abiotic stresses than seedlings obtained from un-primed seeds [56,58,59]. Looking at the importance of bacterial endophytes in plant health, the present study was undertaken with the objective to explore the impact of seed biopriming with microbial inoculant on the induction of local and systemic defense responses to *R. solani*, a necrotrophic fungus causing banded sheath and leaf blight in maize. In this manuscript, we discussed the recent advances for understanding the biochemical and molecular mechanisms employed by *P. aeruginosa,* an endophytic plant symbiont, as a potential biocontrol agent under pathogenic stress.

## 2. Materials and Methods

### 2.1. Characterization of Pseudomonas aeruginosa MF-30

*Pseudomonas aeruginosa* strain MF-30 (NCBI GenBank Accession No. MH177243) isolated from maize rhizosphere, was obtained from Plant-Microbe Interaction and Rhizosphere Biology Lab, ICAR-National Bureau of Agriculturally Important Microorganisms, Kushmaur, Maunath Bhanjan (India). During the course of investigation, *P. aeruginosa* MF-30 was screened for its antimicrobial potential as per methods described by Singh et al. [39]. Briefly, the test strain MF-30 was streaked at the edge of a Petri dish containing potato dextrose agar medium (HiMedia, Mumbai, India). Further, a mycelial plug of *R. solani* (5 mm) was placed at the center of the same plates. The Petri dishes were sealed with a parafilm strip (Tarson, Kolkata, India) and incubated at 27 ± 1 °C for 5 days. Petri dishes with mycelial plug of test pathogen without bacterial culture at the edge were taken as control. In crude extract experiments, *P. aeruginosa* MF-30 was inoculated into nutrient broth (HiMedia, Mumbai, India) and incubated at 27 ± 1 °C for 72 h. After 72 h of incubation, the broth was centrifuged for 10 min at 10,000 rpm. The supernatant was collected in sterilized tubes and filtered with the help of a syringe filter (pore size 0.22 μm). Filter-sterilized culture filtrate was evaluated by employing the agar well diffusion method [39]. To see the effects of volatile organic compounds produced by MF-30 on the inhibition of mycelial growth, an inverted plate assay was performed as per methods described by Singh et al. [39]. The percentage value of inhibition was calculated against the control (untreated) plate at 5 days of inoculation at 27 ± 1 °C under controlled laboratory conditions. For each treatment, there were five replicates (Petri dishes) and the experiment was repeated thrice. Further, strain MF-30 was screened for plant growth-promoting traits, such as phosphate solubilization, potash solubilization, zinc solubilization, indole acetic acid (IAA), hydrogen cyanide (HCN), ammonia, and siderophore production under in vitro conditions. Briefly, phosphate solubilization was assessed by inoculating broth culture of MF-30 on NBRI-P medium, and development of a clear hallow zone around the bacterial colony indicated phosphate solubilizing capability [60]. Potash solubilizing capability was detected by modified Aleksandrov method as described elsewhere [61] using acid–base indicator dye bromothymol blue.

Zinc solubilization was assessed by inoculating MF-30 onto tris mineral medium enriched with zinc oxide, zinc carbonate, and zinc phosphate as described elsewhere [62]. Development of a clear zone around the colonies indicated capability of MF-30 for solubilization of insoluble zinc sources. Ammonia production by the MF-30 was tested as per the protocol described elsewhere [63]. Culture was inoculated in peptone water and allowed to grow at 28 ± 1 °C for a period of 48–72 h followed by addition of 0.5 mL of Nessler’s reagent. Development of yellow-brown color indicated positive reaction for ammonia production. Hydrogen cyanide (HCN) was assessed as per the protocol of Ahmad et al. [64]. MF-30 was inoculated on to the nutrient agar medium amended with glycine (4.4 g L^−1^) and a filter paper soaked in 2% sodium carbonate in 0.5% picric acid solution was placed on the upper lids of Petri plates. The sealed plates were incubated at 28 ± 1 °C for 6 days, and a change in color to orange-brown indicated positive reaction for HCN production. The iron chelating ability by siderophore production was assessed using a modified chrome azurol S (CAS) agar method as described by Schwyn and Neilands [65]. MF-30 culture was spotted on to CAS agar plates and incubated for 3 days at 28 ± 1 °C. Development of an orange halo indicated siderophore production by MF-30. *P. aeruginosa* MF-30 was also screened for the production of hydrolytic enzymes such as amylase, cellulase, pectinase, and chitinase using the standard protocols. Briefly, actively grown culture of MF-30 was inoculated on starch agar plates in order to assess starch hydrolysis by amylase production and incubated at 28 ± 1 °C for 48 h. After incubation, the plate was flooded with iodine solution for a period of 30 s. Development of a clear halo at the edges of colonies indicated amylase production [66].

Cellulase production by MF-30 was assessed on the minimal medium supplemented with 1% carboxy methyl cellulose (CMC). After 48 h of incubation, plates were flooded with Congo red (0.1%) for 20 min and thereafter washed with 1 M NaCl (15 min). Appearance of an orange zone around the colony was considered positive for cellulase production [67]. Chitinase production by MF-30 was detected by inoculating culture in the agar medium added with colloidal chitin. The medium consisted of Na_2_HPO_4_ (6 g L^−1^), KH_2_PO_4_ (3 g L^−1^), NH_4_Cl (1 g L^−1^), NaCl (0.5 g L^−1^), yeast extract (0.05 g L^−1^), and agar (15 g L^−1^) enriched with colloidal chitin 1% (*w*/*v*). Appearance of a clear zone around the colony was considered positive for chitinase production [68]. Pectinase production was assessed by inoculating MF-30 culture in minimal medium supplemented with pectin and incubated at 28 ± 1 °C for 96 h. Following the incubation, the plates were flooded with 0.1% Congo red for 20 min and washed with 1 M NaCl (15 min). Development of an orange clearing zone around the bacterial colony indicated pectinase activity [69].

### 2.2. Green Fluorescent Protein (GFP)-Tagging and Root Colonization Assay

To study the root colonization and epiphytic survival of the test organism, *P. aeruginosa* strain MF-30 was tagged with Green fluorescent protein (GFP) and visualized under a confocal scanning laser microscope (Nikon, Japan) as per methods described by Singh et al. [43]. Briefly, the mini-Tn5 gusA:gfp cassette was inserted into *P. aeruginosa* MF-30 by triparental mating with *E.coli* S17–1 containing Tn5 gusAgfp cassette and *E. coli* HB101 harboring pRK2013. Trans-conjugants were selected on Luria Broth (LB) Agar containing nalidixic acid and kanamycin. The confirmation of GFP tagging of clones was done through colony PCR amplification of the desired amplicon. To select the GFP-tagged mutants, confocal microscopy was done. During examination under UV light, the GFP-tagged mutant showed prominent fluorescence as compared to wild type (Supplementary Figure S1), which indicated that tagging was done properly and GFP expression took place. Based on relative fluorescent activities of a mutant, the best mutant of GFP-tagged *P. aeruginosa* MF-30 was selected and used in further experiments.

Further, maize seeds were bio-primed with GFP-tagged *P. aeruginosa* MF-30 and sown in the pot containing sterile sand–soil mixture (sand:soil:vermiculite in 1:1:1 ratio). Fifteen days after sowing, plants were up-rooted gently, washed in running tap water and microscopy was done as per the methods described by Singh et al. [70]. Briefly, confocal imaging was done using 488 and 543 nm channels under a confocal scanning laser microscope (Nikon Eclipse Confocal A1, Japan). Images were acquired and processes using NIS element software (Nikon, Japan).

### 2.3. Nethouse Experiments

#### 2.3.1. Preparation of Liquid Formulation

The liquid-based formulation of *P. aeruginosa* MF-30 was developed using nutrient broth constituent as a base (the composition of the nutrient broth was: peptone—5 g, meat extract—3 g; sodium chloride—8 g; water 1000 mL; pH at 25 °C—7.3 ± 0.2). Briefly, nutrient broth was prepared, autoclaved, and inoculated with GFP-tagged *P. aeruginosa* MF-30. The inoculated flasks were incubated in a shaking incubator (RPM 150) at 28 °C. After 72 h of growth, 10% sterile glycerol along with 0.01% PVP was added to the nutrient broth. The colony forming unit (CFU) of the end product was calculated using serial decimal dilution method. The CFU count of the liquid formulation was 3.25 × 10^8^ mL^−1^. For foliar spray, the formulation was diluted by adding sterile water and maintained 2 × 10^6^ CFU mL^−1^. The effect of culture filtrate on disease development was studied under nethouse conditions. For this, culture filtrate of *P. aeruginosa* MF-30 was extracted. To extract the culture filtrate, broth was prepared as mentioned above, inoculated with *P. aeruginosa* MF-30 and incubated in a shaking incubator (RPM 150) at 28 °C for 120 h following the methods of Singh et al. [39]. The culture suspension and culture filtrate were applied as foliar application at the rate of 5 mL per plants during evening hours (16:00 h) to avoid direct effects of sunlight.

#### 2.3.2. Planting Material and Growth Conditions

Maize seeds (cv. Sachin 777) were purchased from open market, Maunath Bhanjan, Uttar Pradesh, India. Surface sterilization was done with sodium hypochlorite (NaOCl, 1%) for 2 min, followed by three washing cycles with sterile distilled water under aseptic conditions. Maize seeds were bio-primed with liquid formulation of *P. aeruginosa* MF-30 (10 mL kg^−1^ seed suspended in 40 mL of water containing 0.01% gum acasia, 1.25% chitoson, and 0.01% trehalose), incubated overnight under the shade and sown in pots containing sterile sand–soil mixture (5 kg) during evening hours (16:00 h). Seeds treated with sterile nutrient broth containing 10% sterile glycerol along with 0.01% PVP served as control (10 mL kg^−1^ seed suspended in 40 mL of water containing 0.01% gum acasia, 1.25% chitoson, and 0.01% trehalose). The experiments were laid out during July–October with 75%–90% relative humidity under 11/13 h light/dark photoperiod. Moisture content (at field capacity, 60%) in the pots was maintained by sprinkling sterilized water on every alternate day.

#### 2.3.3. Experimental Set-Up

The experimental design comprised six different treatments in five replications. The treatments were: T_1_-plants inoculated with *R. solani* alone, T_2_-*R. solani* + seed bio-primed with *P. aeruginosa* MF-30; T_3_-*R. solani* + seed bio-primed with *P. aeruginosa* MF-30 + foliar spray of MF-30; T_4_-*R. solani* + foliar spray of culture filtrate of MF-30; T_5_-*R. solani* + seed bio-primed with *P. aeruginosa* MF-30 + foliar spray of culture filtrate of MF-30; T_6_-Control (untreated). The experiments were set up as per treatments in a randomized block design (RBD) under nethouse conditions. Each pot containing 5.0 kg of sand–soil mixture and two plants were maintained in each pot under nethouse conditions. After 30 days of sowing, plants were used for further pathogen inoculation and subsequent experimentations. The foliar spray of MF-30 and culture filtrate was done at 24 h of pathogen inoculation.

#### 2.3.4. Effects of Seed Bio-Priming on the Accumulation of Defense-Related Biomolecules and Enzymes

Maize leaves were sampled from each treatment to measure the total chlorophyll and total carotenoids at 15 days after pathogen inoculation (DAPI). The leaf and root samples were collected and brought to the laboratory. Total chlorophyll and total carotenoids content were measured as per methods described by Sadasivam and Manickam [71]. However, total soluble sugar, total protein, proline, and H_2_O_2_ content in the maize leaves and roots was estimated as per methods given by Thimmaiah [72] at 7 DAPI.

The quantitative estimation of phenylalanine ammonia lyase (PAL), ascorbate peroxidase (APx), peroxidase (POx), and catalase (CAT) was done as per methods described by Sadasivam and Manickam [71] at 7 DAPI. Briefly, to estimate the activity of phenylalanine ammonia lyase (PAL), we ground the tissue sample (1 g) in 4 mL 0.2 M borate buffer (pH 8.7) with 1.4 mM β-mercaptoethanol. The enzyme extract (200 µL) was used for assay using L-phenylalanine and cinnamic acid as substrate, and it was determined spectrophotometrically at 290 nm. For peroxidase activity, enzyme extract (200 µL) was used with 20 mM guaiacol and 12.3 mM H_2_O_2_, and absorbance was recorded at 436 nm every 30 s for 3 min. Further, to estimate the ascorbate peroxidase, enzyme extract was prepared and ascorbic acid (10 mM) was added as substrate, and the change in absorbance was recorded at 265 nm every 30 s for 5 min. Catalase activity was determined using 2.5 mM H_2_O_2_ and enzyme extract. The activity was measured by monitoring the degradation of H_2_O_2_ by spectrophotometer at 240 nm for 1 min. The chitinase and superoxide dismutase (SOD) activity were analyzed in the plant leaves and roots following the methods of Thimmaiah [72].

#### 2.3.5. RNA Extraction and qRT–PCR

Total RNA was extracted from maize leaf and root samples using RNA isolation kit (Agilent, USA) according to the manufacturer’s protocols at 3 and 7 DAPI. cDNA synthesis was done using cDNA synthesis kit (Bio-Rad, USA) following the manufacturer’s protocols. qRT–PCR was performed with a SYBR qPCR Mix kit (Agilent, USA) in a Bio-Rad RT-PCR (MJ MiniOpticon™). *ZmACTIN* (a housekeeping gene) was used to normalize all qRT–PCR data. The primer sequences used in the qPCR analysis are listed in Table 1. The relative expression of *ZmPR-1* and *ZmPR-10* was calculated using the 2^−ΔΔCt^.

#### 2.3.6. Effects of Seed Bio-Priming on Disease Severity and Plant Growth

Five plants of each treatment were sampled randomly. To study the colonization and infection process, plant leaves inoculated with *R. solani* alone were sampled, stained with phloxin-B and visualized under confocal scanning laser microscope using 488 and 543 nm laser line. Further, the selected plant samples were taken to measure the disease severity (%) and lesion length (cm) at 15 and 30 DAPI. The average fresh and dry weight of shoot and root was measured at 30 DAPI.

### 2.4. Statistical Analyses

The controlled laboratory experiments were carried out in a completely randomized design (CRD) in five replications; however, nethouse experiments were laid out in a randomized block design (RBD) in five replications. Data were subjected to analysis of variance and least significant difference (LSD) at *p* < 0.05 using the Statistical Package for Social Sciences Version 16.0 program (SPSS Inc., Chicago, IL, USA, 2007). Data were compared with Duncan’s multiple range test at *p* < 0.05. Graphs were prepared using Microsoft Office Excel 2010 (Microsoft, Washington, DC, USA).

## 3. Results

### 3.1. Characterization of Pseudomonas aeruginosa MF-30

**Figure 1:**
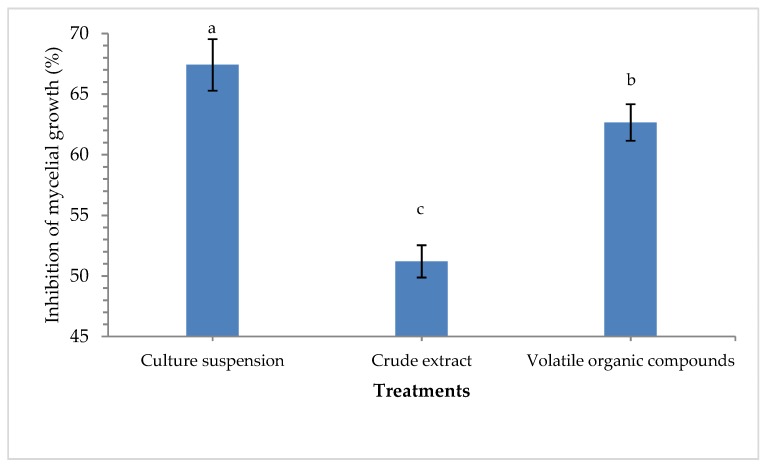
Percent inhibition of *R. solani* mycelia by culture suspension, crude extract, and volatile organic compound produced by *P. aeruginosa* MF-30 at 5 days of inoculation in dual plate assay.

*Pseudomonas aeruginosa* strain MF-30 used in this study showed prominent antagonistic reactions against test pathogens *R. solani* on the dual plate. Results showed that maximum mycelial growth suppression of *R. solani* was recorded in the culture suspension (67.41%) followed by volatile organic compounds (62.66%) as compared to crude extract (51.20%) in a dual plate assay at 5 days of inoculation (Figure 1). *P. aeruginosa* strain MF-30 was found positive for phosphate, potash, and zinc solubilization on respective media under controlled laboratory conditions. It was also found to produce IAA, HCN, siderophore, and ammonia. Further, *P. aeruginosa* MF-30 was screened for the production of hydrolytic enzymes *viz.* amylase, cellulase, pectinase, and chitinase, and qualitative estimation showed that the test endophyte was producing these enzymes in the plate assay (Supplementary Table S1 and Supplementary Figure S2).

### 3.2. GFP-Tagging and Root Colonization Assay

**Figure 2:**
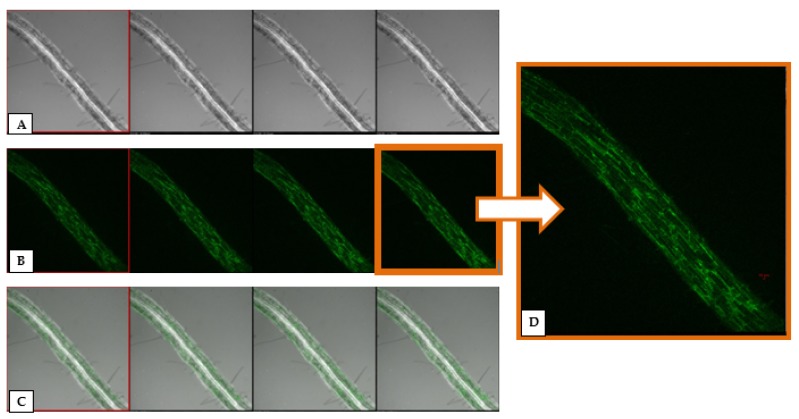
Z-stack of maize root visualized under confocal scanning laser microscopy indicating localization of Green fluorescent protein (GFP)-tagged *Pseudomonas aeruginosa* MF-30 in the maize roots (10×). (**A**) Z-stack of different planes from Transmission Detector (TD) channel, (**B**) Z-stack of different planes from 488 nm channel, (**C**) Z-stack of different planes from both TD and 488 nm channel, and (**D**) magnified X–Y plane image of maize roots showing colonization of MF-30 by GFP fluorescence.

To investigate whether *P. aeruginosa* MF-30 has the potential to colonize root system or not, an experiment was conducted under nethouse conditions. Confocal microscopic observations clearly indicated that *P. aeruginosa* MF-30 colonizes the entire root (Figure 2). More than 80% roots were showing bacterial signal and it was also confirmed by re-isolation of tagged bacteria from different parts of roots. It was clearly shown that *P. aeruginosa* MF-30 colonizes internally in the cortical tissues, endodermis, bundle sheath, and vascular bundles (xylem and phloem). Further, *P. aeruginosa* MF-30 colonization did not result in any morphological changes in the root anatomy, growth, and development pattern.

### 3.3. Effects of Seed Bio-Priming on the Accumulation of Chlorophyll and Carotenoids Content

**Figure 3:**
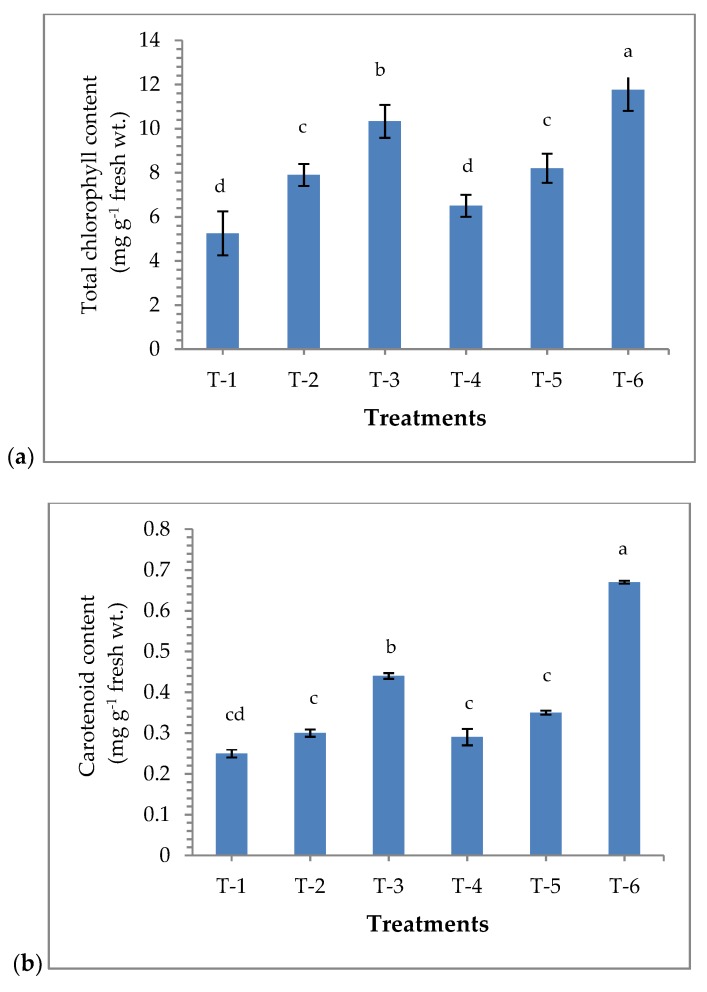
Effects of seed bio-priming and other treatments on (**a**) total chlorophyll content and (**b**) total carotenoids content in maize leaves at 15 DAPI under nethouse condition. Treatments were: T_1_-plants inoculated with *R. solani* alone; T_2_-*R. solani* + seed bio-primed with *P. aeruginosa* MF-30; T_3_-*R. solani* + seed bio-primed with *P. aeruginosa* MF-30 + foliar spray of MF-30; T_4_-*R. solani* + foliar spray of culture filtrate of MF-30; T_5_-*R. solani* + seed bio-primed with *P. aeruginosa* MF-30 + foliar spray of culture filtrate of MF-30; T_6_-Control (untreated). Data are mean ± SEM (*n* = 5).

Results revealed that maize plants bio-primed with *P. aeruginosa* MF-30 have significant effect on the chlorophyll content in the maize plants. A significantly higher amount of chlorophyll was recorded in the plants bio-primed with *P. aeruginosa* MF-30 and subsequent foliar spray of MF-30 culture suspension (10.33 g^−1^ fresh wt.), followed by plants bio-primed with *P. aeruginosa* MF-30 and subsequent foliar spray of culture filtrate (8.20 g^−1^ fresh wt.), as compared to other treatments under pathogenic stress of *R. solani*. However, maximum chlorophyll content was recorded in uninoculated control plants (11.75 g^−1^ fresh wt.), while the lowest amount of chlorophyll was recorded in *R. solani* (alone) challenged plants (5.25 g^−1^ fresh wt.) at 15 DAPI (Figure 3a). A similar pattern was recorded for the total carotenoids content in the plant bio-primed with *P. aeruginosa* MF-30 under pathogenic challenged at 15 DAPI (Figure 3b).

### 3.4. Effects of Seed Bio-Priming on Total Soluble Sugar, Protein, Proline, and H_2_O_2_ Content

**Figure 4:**
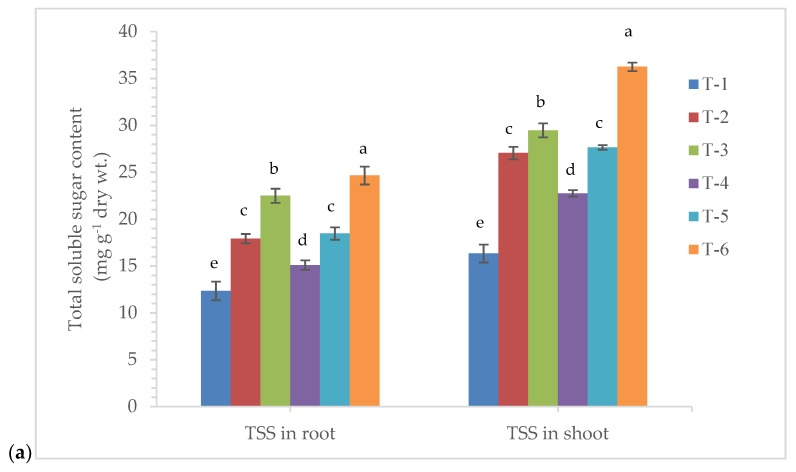

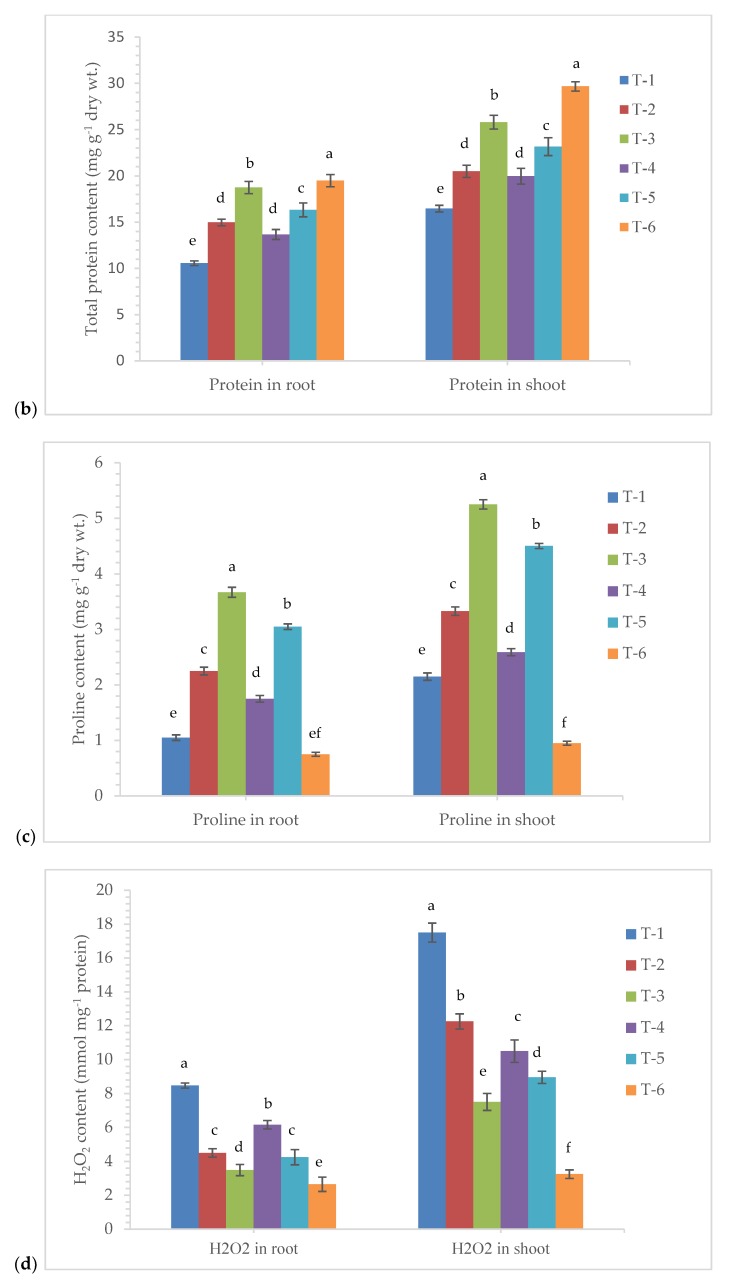
Effects of seed bio-priming and other treatments on (**a**) total soluble sugar content, (**b**) total protein content, (**c**) total proline, and (**d**) H_2_O_2_ content in maize roots and shoot at 7 DAPI under nethouse condition. Treatments were: T_1_-plants inoculated with *R. solani* alone; T_2_-*R. solani* + seed bio-primed with *P. aeruginosa* MF-30; T_3_-*R. solani* + seed bio-primed with *P. aeruginosa* MF-30 + foliar spray of MF-30; T_4_-*R. solani* + foliar spray of culture filtrate of MF-30; T_5_-*R. solani* + seed bio-primed with *P. aeruginosa* MF-30 + foliar spray of culture filtrate of MF-30; T_6_-Control (untreated). Data are mean ± SEM (*n* = 5).

Changes in the accumulation and content of the biomolecules show the real impact of treatments in the plants. To assess the effects of endophytic *P. aeruginosa* MF-30 on the accumulation of total soluble sugar, protein, proline and H_2_O_2_ content in maize, we measured these compounds in the plant leaves and roots at 7 DAPI. Quantitative estimation indicated that a significantly higher amount of total soluble sugar was recorded in the roots and leaves of the plants bio-primed with *P. aeruginosa* MF-30 and subsequent foliar spray of culture suspension of MF-30 (22.50 and 29.47 mg g^−1^ dry wt., respectively), followed by plants bio-primed with *P. aeruginosa* MF-30 and subsequent foliar spray of culture filtrate (18.46 and 27.66 mg g^−1^ dry wt., respectively) and plants bio-primed with *P. aeruginosa* MF-30 (17.92 and 27.05 mg g^−1^ dry wt., respectively) at 7 DAPI (Figure 4a).

Seed bio-priming with *P. aeruginosa* MF-30 significantly affected the total protein content in the maize plants and enhancement of protein content was also evident from increased plant growth parameters. Plants bio-primed with *P. aeruginosa* MF-30 and foliar spray of culture suspension of MF-30 in combination showed significant (*p* < 0.05) increase in the total protein content in the maize root (17.75 mg g^−1^ dry wt.) and leaves (25.79 mg g^−1^ dry wt.) at 7 DAPI under pathogenic stress conditions (Figure 4b). However, the least amount of protein was measured in the roots and shoot of the plants treated with *R. solani* alone (10.56 and 16.47 mg g^−1^ dry wt.) at 7 DAPI (Figure 4b).

Proline plays an important role in plant defense and signaling under pathogenic stress conditions. In the present study, results indicated that bacterial endophyte colonizes the internal root tissues and trigger proline synthesis in the maize root and shoot. The proline production was in the range of 0.75–5.25 mg g^−1^ dry wt. It was found that plants bio-primed with strain MF-30 and subsequent foliar spray of culture suspension of MF-30 expressed the maximum amount of proline in the root (3.67 mg g^−1^ dry wt.) and shoot (5.25 mg g^−1^ dry wt.) pre-challenged with *R. solani* at 7 DAPI followed by plants bio-primed with strain MF-30 and foliar spray of culture filtrate (Figure 4c). However, the least proline content was recorded in the control plant.

The quantitative estimation of H_2_O_2_ was done to see the effects of *R. solani* infection on the maize plant. In our results, a sharp increase in the amount of H_2_O_2_ was recorded in the plants pre-challenged with *R. solani* at 7 DAPI, and it was reported to be maximum in the *R. solani* alone treated plant root (8.47 mmol mg^−1^ protein) and shoot (17.50 mmol mg^−1^ protein). It was observed that H_2_O_2_ concentration sharply decreased in the plants bio-primed with *P. aeruginosa* MF-30 and foliar spray with culture suspension and culture filtrate (Figure 4d). It was greatly reduced in the plants bio-primed with *P. aeruginosa* MF-30 and foliar spray with culture suspension of MF-30, which revealed the application of *P. aeruginosa* MF-30 efficiently reduced the stress in the plant system. However, the least amount of H_2_O_2_ was recorded in the root (2.65 mmol mg^-1^ protein) and shoot (3.25 mmol mg^−1^ protein) of untreated control plants at 7 DAPI. It was observed that the concentrations of total soluble sugar, total protein, total proline, and H_2_O_2_ were significantly higher in shoots as compared to roots across the treatments.

### 3.5. Effects of Seed Bio-Priming on Antioxidant Enzymes Activity

**Figure 5:**
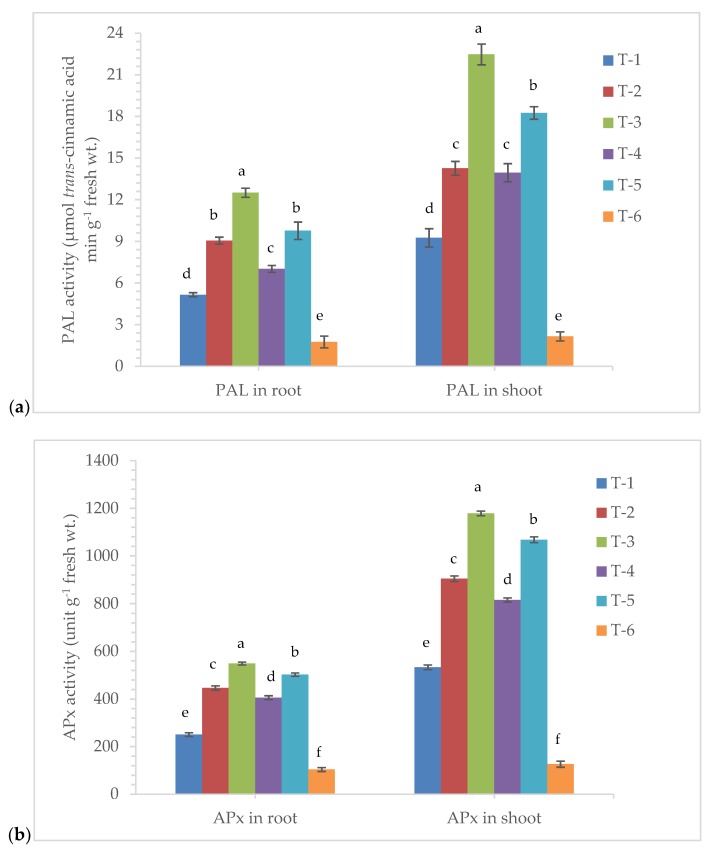

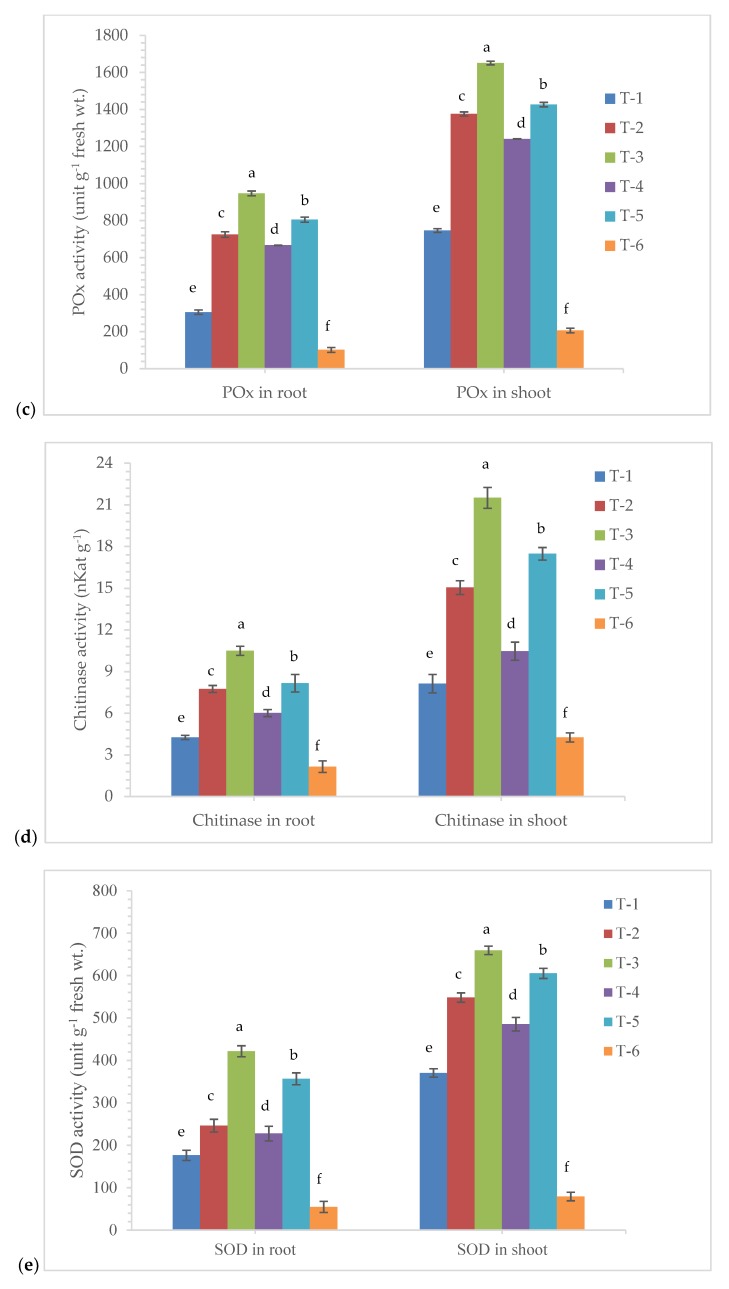

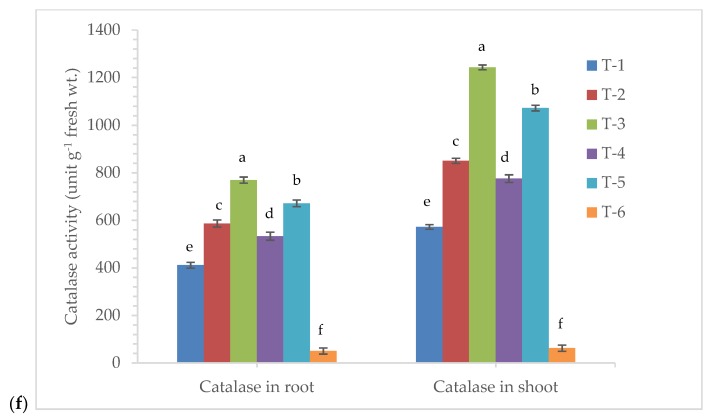
Effects of seed bio-priming and other treatments on activity of antioxidant enzymes (**a**) phenylalanine ammonia lyase (PAL), (**b**) ascorbate peroxidase (APx), (**c**) peroxidase (POx), (**d**) chitinase, (**e**) superoxide dismutase (SOD), and (**f**) catalase (CAT) activity in maize root and shoot at 7 DAPI under nethouse condition. Treatments were: T_1_-plants inoculated with *R. solani* alone; T_2_-*R. solani* + seed bio-primed with *P. aeruginosa* MF-30; T_3_-*R. solani* + seed bio-primed with *P. aeruginosa* MF-30 + foliar spray of MF-30; T_4_-*R. solani* + foliar spray of culture filtrate of MF-30; T_5_-*R. solani* + seed bio-primed with *P. aeruginosa* MF-30 + foliar spray of culture filtrate of MF-30; T_6_-Control (untreated). Data are mean ± SEM (*n* = 5).

The bio-primed plants tended to overproduce defense-related biomolecules and antioxidant enzymes under pathogenic stress of *R. solani* in maize at 7 DAPI. The phenylalanine ammonia lyase (PAL) is the key enzyme of phenylpropanoid pathway and plays an important role in the plant defense during pathogen infection. The amount of PAL produced and accumulated in the bio-primed plant was found significantly increased after pathogen inoculation and/or exposer to the phytotoxin produced by them in the leaf tissues. It was reported to be significantly higher in all the treatments compared to plants treated with *R. solani* alone and untreated control plants at 7 DAPI (Figure 5a). The maximum activity of PAL was recorded in the plant root (12.50 µmol *trans*-cinnamic acid min g^−1^ fresh wt.) and shoot (22.47 µmol *trans*-cinnamic acid min g^−1^ fresh wt.) bio-primed with *P. aeruginosa* MF-30 and foliar spray with culture suspension of MF-30 followed by plants bio-primed with strain MF-30 and subsequent spray with culture filtrate of MF-30, compared to pathogen alone treated plants (5.15 and 9.25 µmol *trans*-cinnamic acid min g^−1^ fresh wt., respectively). However, the least activity of PAL was recorded in the root (1.75 µmol *trans*-cinnamic acid min g^−1^ fresh wt.) and shoot (2.15 µmol *trans*-cinnamic acid min g^−1^ fresh wt.) of the untreated control plants (Figure 5a).

Ascorbate peroxidase (APx) is supposed to be an efficient scavenger of H_2_O_2_ and O_2_^−^ which shows that APx has a much higher affinity to H_2_O_2_ and O_2_^−^ under pathogenic stress conditions. The APx activity was reported to be highest in the root (549.16 unit g^−1^ fresh wt.) and shoot (1178.25 unit g^−1^ fresh wt.) of the plants bio-primed with *P. aeruginosa* MF-30 and foliar spray with culture suspension of MF-30 followed by plant bio-primed with strain MF-30 and subsequent spray with culture filtrate of MF-30 and plants bio-primed with *P. aeruginosa* MF-30, compared to pathogen alone treated plants (Figure 5b).

Peroxidase (POx) is one of the most important enzymes activated during biotic and abiotic stress tolerance, and it is considered the final enzyme in the monolignol biosynthesis pathway/phenylpropanoid pathway. The level of POx varies significantly and is dependent on the type and extent of stress. In the present study, it ranged from 101.50 to 1650 unit g^−1^ fresh wt. The quantitative measurement of POx indicated that a significantly higher amount of POx activity was recorded in the root (947.15 unit g^−1^ fresh wt.) and shoot (1650.46 unit g^−1^ fresh wt.) of the plants bio-primed with *P. aeruginosa* MF-30 and subsequent foliar spray of culture suspension of MF-30 followed by bio-primed with *P. aeruginosa* MF-30 and subsequent foliar spray of culture filtrate of MF-30 and plant bio-primed with *P. aeruginosa* MF-30 (Figure 5c). However, the lowest amount of POx was recorded in the untreated control plants and plants treated with *R. solani* alone (Figure 5c). A similar trend was recorded for chitinase activity across the treatments where maximum chitinase activity was recorded in the root (10.50 nKat g^−1^ of plant biomass) and shoot (21.50 nKat g^−1^ of plant biomass) of the plants bio-primed with *P. aeruginosa* MF-30 and subsequent foliar spray of culture suspension of MF-30 at 7 DAPI (Figure 5d).

Further, superoxide dismutase (SOD) and catalase (CAT) are excellent scavengers of superoxide radicals generated during the plant–pathogen interaction process. In the quantitative estimation, significant changes were recorded in the activities of SOD and CAT in the plants bio-primed with *P. aeruginosa* MF-30 and pre-challenged by *R. solani* at 7 DAPI. The maximum activity of SOD was recorded in the root (421.50 unit g^−1^ fresh wt.) and shoot (659.42 unit g^−1^ fresh wt.) of the plants bio-primed with *P. aeruginosa* MF-30 and subsequent foliar spray of culture suspension of MF-30 followed by plants bio-primed with *P. aeruginosa* MF-30 and foliar spray of culture filtrate (356.50 and 605.25 unit g^−1^ fresh wt., respectively) and plants bio-primed with *P. aeruginosa* MF-30 and pre-challenged by *R. solani* (245.96 and 548.19 unit g^−1^ fresh wt., respectively) at 7 DAPI (Figure 5e). A similar trend was recorded for catalase activity in the bio-primed maize pre-challenged with the pathogen (Figure 5f).

### 3.6. Gene Expression Analysis

**Figure 6:**
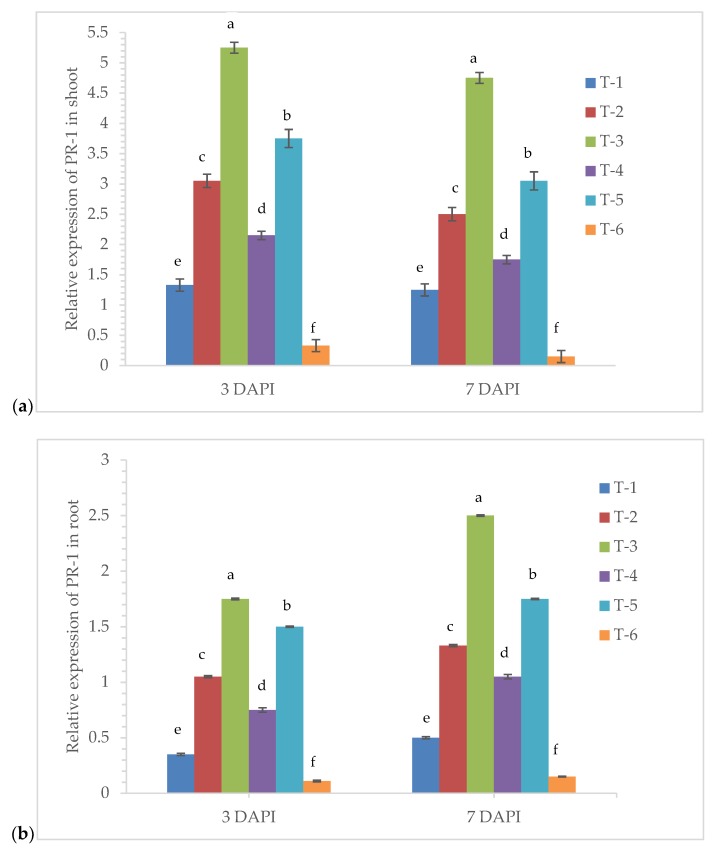

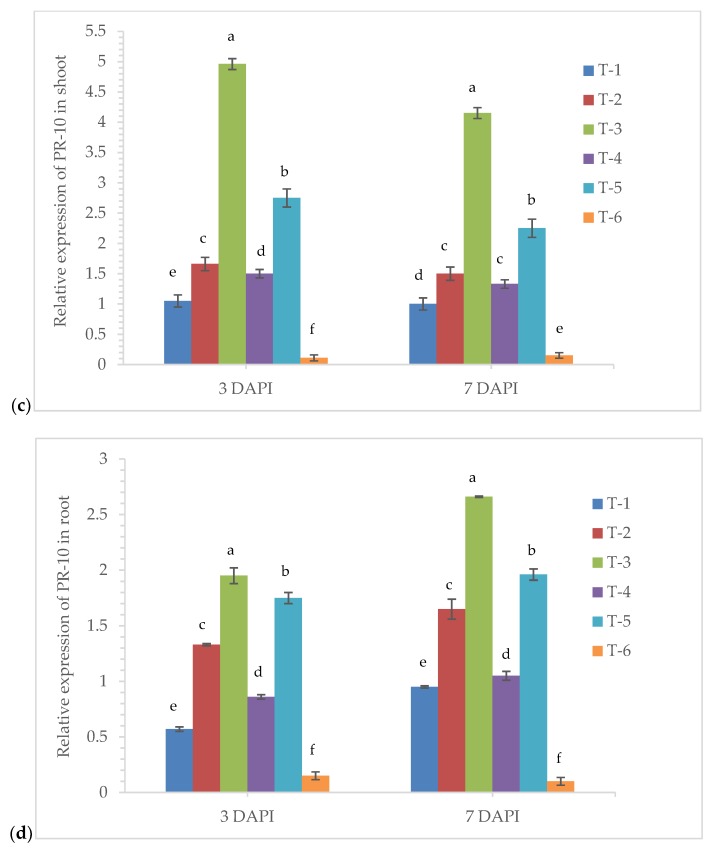
qRT-PCR results showing the time-dependent and tissue-specific differential expression profile of *zmPR-1* and *zmPR-10* in the maize plants bio-primed with *P. aeruginosa* MF-30 at 3 and 7 DAPI, (**a**) relative expression of *zmPR-1* in shoot, (**b**) relative expression of *zmPR-1* in root, (**c**) relative expression of *zmPR-10* in shoot, and (**d**) relative expression of *zmPR-10* in root. Treatments were: T_1_-plants inoculated with *R. solani* alone; T_2_-*R. solani* + seed bio-primed with *P. aeruginosa* MF-30; T_3_-*R. solani* + seed bio-primed with *P. aeruginosa* MF-30 + foliar spray of MF-30; T_4_-*R. solani* + foliar spray of culture filtrate of MF-30; T_5_-*R. solani* + seed bio-primed with *P. aeruginosa* MF-30 + foliar spray of culture filtrate of MF-30; T_6_-Control (untreated). The data represents the relative fold changes in the expression value of the six different treatments. Data are mean ± SEM (*n* = 5).

The qRT-PCR analysis unraveled the temporal and tissue-specific expression of pathogenesis-related genes specifically *zmPR-1* and *zmPR-10* in the root and shoot of maize plant bio-primed with endophyte *P. aeruginosa* MF-30. In the present study, we have analyzed the time-dependent and tissue-specific responses in terms of gene expression profile (mRNA transcripts) changes in the maize plants. The quantitative expression of *zmPR-1* and *zmPR-10* was measured in terms of relative fold change in the root and shoot and compared with the untreated control plants at 3 and 7 DAPI. In the qRT-PCR analysis, we saw the up-regulation of *zmPR-1* and *zmPR-10* in the root and shoot tissues (Figure 6a–d). The highest expression of *zmPR-1* was recorded in the shoot of the plants bio-primed with *P. aeruginosa* MF-30 and subsequent foliar spray of culture suspension of MF-30 (5.25-fold) followed by plants bio-primed with *P. aeruginosa* MF-30 and foliar spray of culture filtrate (3.75-fold) and plants bio-primed with *P. aeruginosa* MF-30 and pre-challenged by *R. solani* (3.05-fold) at 3 DAPI (Figure 6a). However, a slight decreased in the expression of *zmPR-1* was recorded at 7 DAPI (Figure 6a). Moreover, the expression of *zmPR-1* in the maize root was recorded to be comparatively less than in the shoot (Figure 6b). However, the trend was similar to the shoot at 3 and 7 DAPI. The lowest level of the transcript was recorded in the root (0.11- and 0.15-fold at 3 and 7 DAPI) and shoot (0.33- and 0.15-fold at 3 and 7 DAPI) of untreated control plants and root (0.35- and 0.50-fold at 3 and 7 DAPI) and shoot (1.33- and 1.25-fold at 3 and 7 DAPI) of the pathogen alone treated plants (Figure 6a,b).

Interestingly, our results clearly showed *P. aeruginosa* MF-30 bio-primed plants had an aggravated defense response as estimated from the transcript profile of *zmPR-1* and *zmPR-10.* The expression profile of *zmPR-10* also followed a more or less similar pattern as recorded in the transcript profile of *zmPR-1* in the maize shoot and root (Figure 6c,d). Comparatively, the higher fold was recorded in the shoot as compared to the root which indicated that expression was tissue- and time-dependent. Though the transcript level was not the same, expression took place locally at the site of infection as well as in the root tissue which clearly indicated that *P. aeruginosa* MF-30 activated the defense responses locally as well as systemically. The activities of defense-related antioxidant enzymes and biomolecule production were also observed.

### 3.7. Epiphytic Colonization of GFP-Tagged P. aeruginosa MF-30 on Maize Leaf

**Figure 7:**
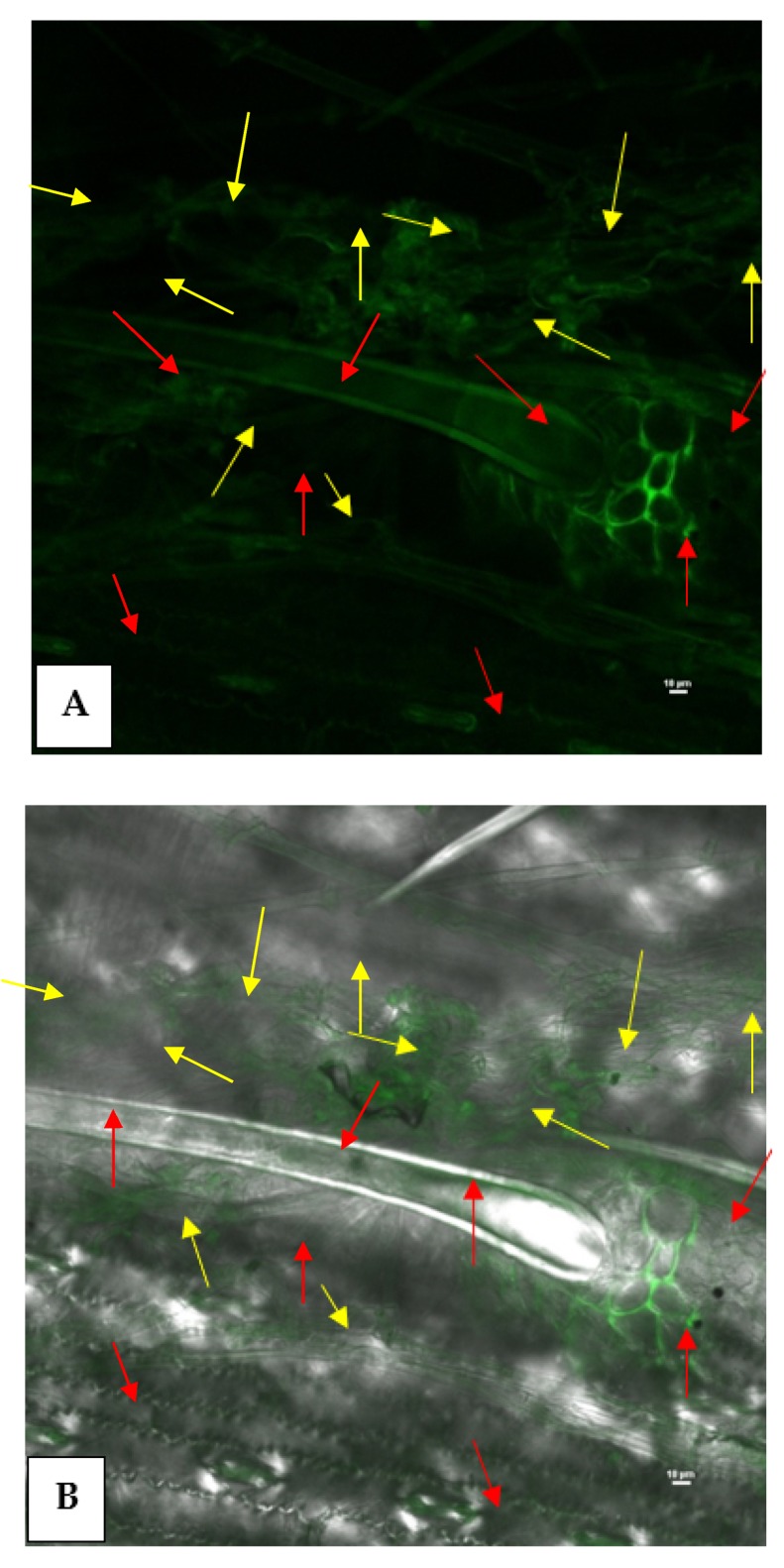
Confocal scanning laser microscopic image showing epiphytic colonization of GFP-tagged *P. aeruginosa* MF-30 on maize leaf. Red arrows indicate the colonization on different parts of the leaf, such as leaf surface, trichomes, basal cells of the trichome, stomata, etc., while yellow arrows show the colonization of *P. aeruginosa* MF-30 on fungal mycelia of *R. solani* and infection cushion developed on maize leaf. (**A**) Confocal micrograph at 488 nm channel and (**B**) confocal micrograph at 488 nm and TD channel together.

Confocal laser microphotographs clearly indicated the epiphytic survival and colonization of *P. aeruginosa* strain MF-30 on the leaf surface. Results indicated that *P. aeruginosa* strain MF-30 colonizes on the fungal mycelia and disintegrates the hyphae and infection cushion formed during the infection process and ultimately restricts the disease development (Figure 7).

**Figure 8:**
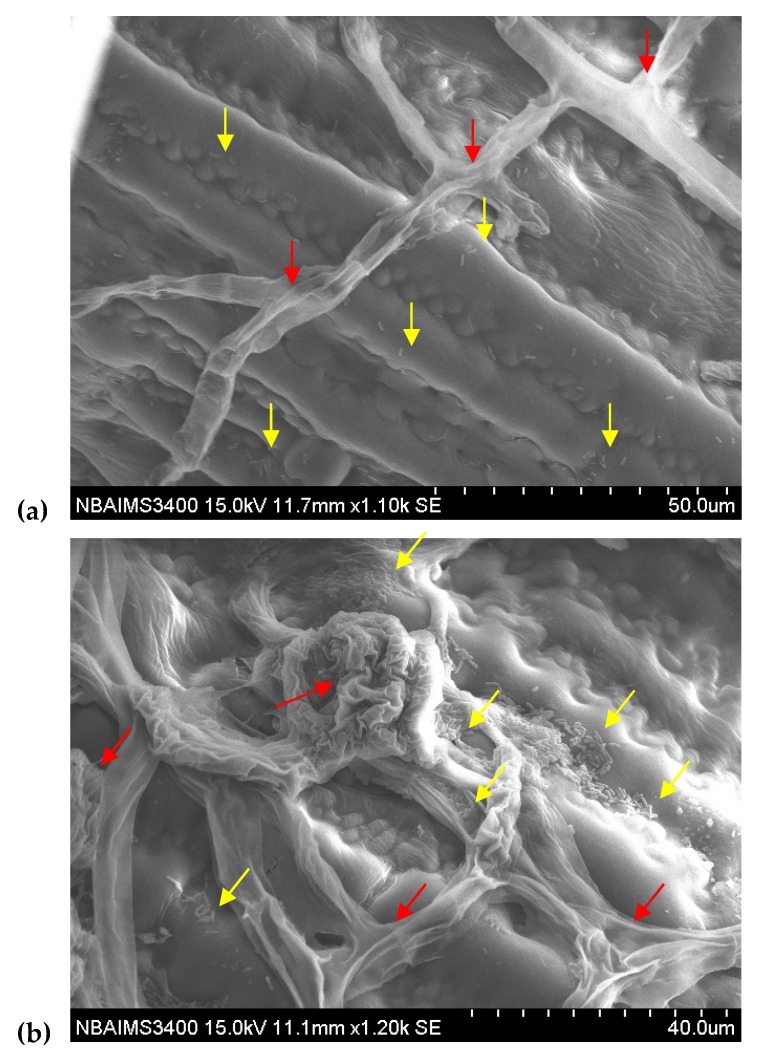
Scanning electron microscopic study showed the epiphytic colonization of GFP-tagged *P. aeruginosa* MF-30 on maize leaf. (**a**) microphotographs taken after 24 h of foliar application, (**b**) microphotographs taken at 7 days of foliar application. Red arrow indicated the fungal mycelia and infection cushion developed by *R. solani* on maize leaf, while yellow arrow showed the colonization of *P. aeruginosa* MF-30 on leaf surface, fungal mycelia, and infection cushion developed on maize leaf. Figure 8 (**b**) clearly indicates the lysis and disintegration of fungal mycelia and infection cushion caused by MF-30.

This was further confirmed by scanning electron microscopy. It was observed that *P. aeruginosa* MF-30 colonizes on the leaf and other parts of the maize plant. The GFP-tagged *P. aeruginosa* MF-30 effectively lysed and disintegrated the infection cushion and invading mycelia of *R. solani* (Figure 8).

### 3.8. Effects of Seed Bio-Priming on Plant Growth and Disease Development

**Figure 9:**
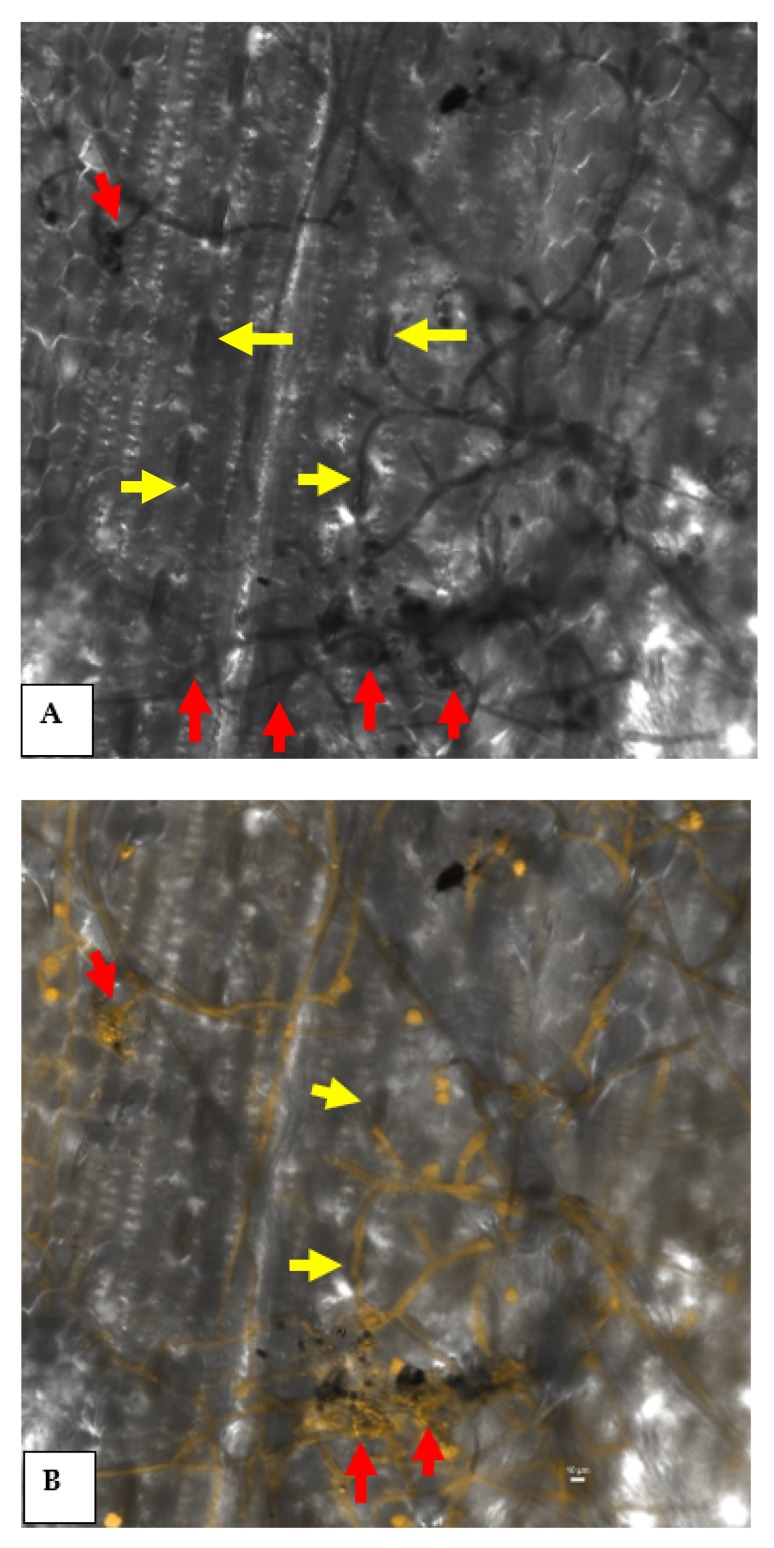
Confocal scanning laser microphotographs showing the colonization and infection of *R. solani* in maize. The red arrows indicate the formation of infection cushion, and yellow arrows indicate the entry of fungal mycelia through stomata. (**A**) Micrograph in TD channel and (**B**) superimposed image of TD and 488 nm channel together.

Seed bio-priming promoted the accumulation of fresh and dry biomass of shoot and root in the pot experiments under nethouse conditions with and without the test pathogen, *R. solani*. Earlier, it was reported that *R. solani* formed an infection cushion to penetrate the plant surface by adding mechanical force. However, in the present study, microscopic observations clearly show that *R. solani* colonizes on the plant sheath and leaves and penetrated in the plant leaves actively by forming infection cushion and passively through the stomata (Figure 9A,B). Results revealed that maximum shoot and root biomass (fresh and dry) was recorded in the untreated control plants followed by plants bio-primed with *P. aeruginosa* MF-30 and subsequent foliar spray of culture suspension of MF-30 then followed by the plants bio-primed with *P. aeruginosa* MF-30 and foliar spray of culture filtrate of MF-30 and plants bio-primed with *P. aeruginosa* MF-30 pre-challenged by *R. solani* at 30 DAPI (Table 2). However, the lowest amount of plant biomass was recorded in pathogen alone challenged plants (Table 2).

**Figure 10:**
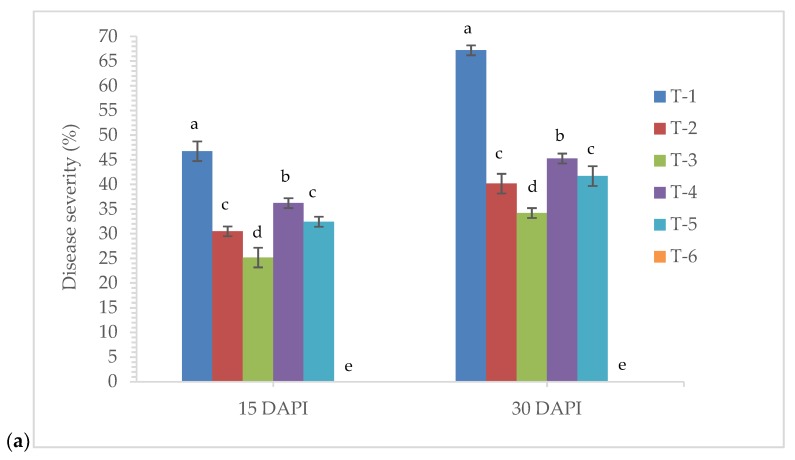

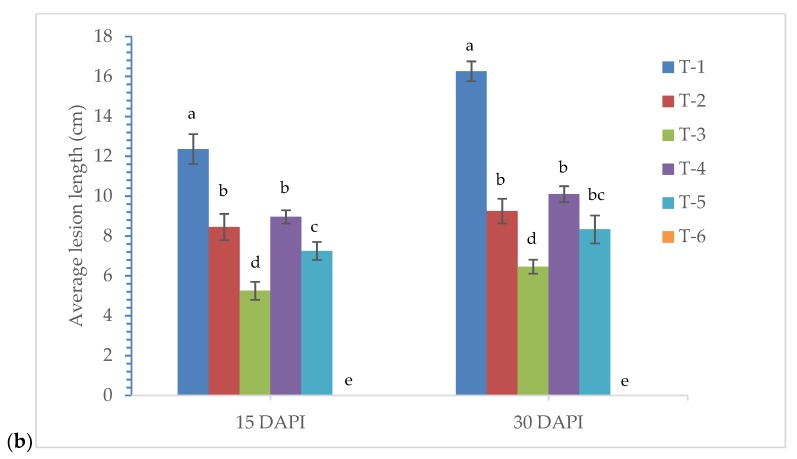
Effects of seed bio-priming on (**a**) disease severity and (**b**) lesion length at 15 and 30 days post inoculation of *R. solani* under nethouse conditions. Treatments were: T_1_-plants inoculated with *R. solani* alone; T_2_-*R. solani* + seed bio-primed with *P. aeruginosa* MF-30; T_3_-*R. solani* + seed bio-primed with *P. aeruginosa* MF-30 + foliar spray of MF-30; T_4_-*R. solani* + foliar spray of culture filtrate of MF-30; T_5_-*R. solani* + seed bio-primed with *P. aeruginosa* MF-30 + foliar spray of culture filtrate of MF-30; T_6_-Control (untreated). Data are mean ± SEM (*n* = 5).

Further, to investigate the effects of seed bio-priming with endophytic *P. aeruginosa* MF-30 and other treatments on lesion length and disease severity at 15 and 30 days post inoculation, nethouse experiments were conducted. Large variations in lesion length and disease severity were recorded in different treatments taken for the study, and the average lesion lengths ranged from 5.25 to 16.25 cm (Figure 10a). However, disease severity ranged from 25.16% to 67.19% (Figure 10b). The maximum disease severity (46.72% and 67.19%, respectively) and lesion length (12.36 and 16.25 cm) were recorded in the plants treated with *R. solani* alone which are significantly higher than other treatments at 15 and 30 days post inoculation. However, minimum disease severity (25.16% and 34.29%, respectively) and lesion length (5.25 and 6.46 cm) were recorded in the plants bio-primed with *P. aeruginosa* MF-30 and subsequent foliar spray of culture suspension of MF-30, followed by the plants bio-primed with *P. aeruginosa* MF-30 and foliar spray of culture filtrate of MF-30 and plants bio-primed with *P. aeruginosa* MF-30 pre-challenged by *R. solani* at 15 and 30 days of post inoculation (Figure 10a,b).

## 4. Discussion

Globally, the occurrence of banded leaf and sheath blight disease caused by *R. solani* is increasing [6] and is now considered one of the most devastating diseases of the *Kharif* maize sown in warm and humid climates. The pathogen *R. solani* becomes more aggressive with an increase in relative humidity at an average temperature of 27–30 °C [1,2,73]. Looking at the importance, the present study was taken to investigate whether seed bio-priming with microbial inoculant triggers local and systemic defense responses against *R. solani* causing banded leaf and sheath blight in maize. In this study, endophytic *P. aeruginosa* MF-30 was taken and characterized for its antimicrobial properties along with various plant growth-promoting traits. Results indicated that *P. aeruginosa* MF-30 was able to inhibit the mycelial growth of *R. solani* up to 67.41% in a dual plate assay using culture suspension. This indicated the possibility of production of water-soluble antimicrobial compounds in MF-30 due to which higher suppression was observed in culture suspension than crude extract. This could further add to the ease of commercial application in order to augment the disease protection. It also possesses various plant growth-promoting traits, such as phosphate, potash, and zinc solubilization, IAA production, etc. Results indicated the biocontrol potential of supplemented MF-30 to show impressive growth promotion characteristics. This provides evidence for the successful inoculation effect of MF-30 to achieve plant growth and health promotion. Tracking of a particular strain in the rhizosphere and/or plant system in an overcrowded environment needs precise and selective detection protocols [17,54,55,56,74]. The selected strain MF-30 was tagged with the GFP and confocal scanning laser microscopy indicated that it could colonize the maize root effectively. During the course of examination under the confocal microscope, the signal of GFP-tagged MF-30 was observed in the coleoptiles at 15 days of inoculation (data not shown). In our previous study, it was reported that *P. geniculata* significantly colonized the epidermis, cortical region, endodermis, and vascular tissues, including xylem, phloem, and bundle sheath [70].

The relationship between the bacterial endophytes and their host plants is well documented [75,76]. Endophytic colonization poses a specific advantage to be adapted in the niche where the biocontrol mechanisms need to be deployed against the pathogen [77]. Understanding epiphytic and endophytic colonization and survival is the pre-requisite for better management of applied inocula [78]. Moreover, the confocal and scanning electron microscopic studies clearly indicated that strain MF-30 successfully colonizes as epiphyte apart from internal colonization. This could further augment the disease protection against BLSB. Bruisson et al. [27] reported that “in general, plants are colonized by a variety of microorganisms, some of which (the epiphytes) stay on the surface of plant organs, while others can penetrate further inside the plants and are called endophytes” [27,28,29]. A recent study on the structure and function of the plant microbiome have focused on the host-mediated recruitment and shaping of their rhizosphere microbiome [42]. These microorganisms play a key role in the plant establishment in a wide range of environmental conditions [38,39,70]. In this, the role of endophytic microorganisms on the plant health is well documented [33].

In several studies, it was reported that plant pathogen interferes with the photosynthetic machinery in the plants. Most of the pathogens produce phytotoxins causing blight symptoms on above ground parts of the plants, especially on leaves, thereby reducing the net photosynthetic area [38,39,43]. Seed bio-priming with MF-30 modulated photosynthetic pathways and significantly reduced the production and accumulation of H_2_O_2_ and ROS in the leaves directly by reducing the pathogen colonization and invasion. Further, during the course of infection, H_2_O_2_ and reactive oxygen species (ROS) were produced in the leaf tissues and causes cell death. It was also reported that genes expression related to photosynthesis and carbon fixation were suppressed by the pathogen infection [79,80]. In the present study, it was observed that chlorophyll and carotenoid content were significantly reduced in the plant leaves treated with *R. solani* alone compared to untreated control and seed bio-primed plants. However, indirect mechanisms involved the production and activity of antioxidant enzymes such as superoxide dismutase, peroxidase, catalase, etc. in the host tissues. These enzymes detoxify the harmful effects ROS and superoxide radicals. In the present study, significantly higher activity of antioxidant enzymes was recorded, which corroborated the findings of several other works [79,80,81]. Further, an increased organic solute content, i.e., total soluble sugar (carbohydrates), proteins, and proline, were found in the leaves and roots of the plants bio-primed with MF-30 under biotic stress of *R. solani*. Organic solutes play a critical role in maintaining the turgor pressure and protecting plants from osmotic stress generated during pathogenic infection [38,39,43]. It was also reported that a small amount of H_2_O_2_ induces the synthesis and accumulation of organic solutes such as carbohydrates, proteins, and proline in the plant tissues under biotic and abiotic stresses [39,82].

These microorganisms colonize the plant system and stimulate plant defense against biotic and abiotic stresses and a phenomenon called induced systemic resistance/tolerance (ISR/IST) [83,84,85]. For the plants bio-primed with beneficial microorganisms and subsequently infected with pathogens, plants employ an arsenal of pattern-recognition receptors (PRRs) that recognize conserved features known as microbe- or pathogen-associated molecular patterns (MAMPs or PAMPs), which activate the pattern-triggered immunity (PTI), the first layer of inducible plant defense [6,86,87,88]. Thereafter, PTI activated the mitogen-activated protein kinases (MAPKs), and the production of reactive oxygen species (ROS) leads to changes in the calcium concentration in the cell. Thus, it finally activates the defense-related genes in the plants and induced systemic resistance takes place [6,89,90,91]. In the present study, significantly higher accumulation and activity of PAL, ascorbate peroxidase, and peroxidase were recorded in the plants bio-primed with *P. aeruginosa* MF-30 and subsequent foliar spray with culture suspension of MF-30, compared to plants inoculated with pathogen alone. These results are in agreement with the other works which demonstrated that PAL is the key enzyme which triggers induced systemic resistance via phenylpropanoid pathway in the plants. The increased level of PAL activated the peroxidase which leads to synthesis of lignin [38]. Further, the PAL activated the synthesis of SA/MeSA, and SA-dependent pathways were activated. SA-dependent disease resistance comprises two pathways—nonexpresser of PR genes 1 (NPR1)-dependent and NPR1-independent pathways [92]. The NPR1-independent pathway directly induces the expression of WRKY transcription factors, while the NPR1-dependent pathway induces the expression of pathogenesis-related (PR) genes via TGA and/or WRKY transcription factors. These elicitors use different routes to activate local and systemic resistance (SAR) in plants against various biotic stressors [93]. However, the activation of plant immune responses depends upon the nature of the pathogen, infection intensity, and host species. In the biotrophic interaction, effector-triggered immunity (ETI) plays a key role, and it activates resistance proteins (R-proteins) in the host [94,95,96]. The R-protein detects pathogen effectors, and hypersensitive response (HR) takes place. This immune response often causes localized cell death, which restricts pathogen spread.

Under biotic stress, more specifically microbial infection, the transcription level of plant genes is up-regulated which code for a specific class of proteins called pathogenesis-related (PR) proteins. The *PR-1* and *PR-10* proteins have been previously reported to have antimicrobial activity and thus play an important role in the plant defense under biotic stress condition [97,98,99]. Further, in the present investigation, the expression of *PR-1* and *PR-10* was found to increase in the plants bio-primed with endophyte *P. aeruginosa* MF-30 and pre-challenged with *R. solani*.

Plant growth-promoting beneficial endophytic bacteria promoted plant growth directly and/or indirectly [33]. Endophytes produce different growth regulators in the plants under normal and stressed conditions. These molecules activated several pathways in the plants which led to better plant growth and development. It was shown that MF-30 produces IAA (Supplementary Table S1 and Supplementary Figure S2). The small amount of IAA activated the auxin-inducible GH3.35–6 which inactivates the JA pool in the elongated hypocotyl and subsequently diminishes the availability of JA-Ile, the bioactive form that triggers JA signaling. JAs promote lateral root initiation by inducing the expression of ERF109 and inhibit primary root elongation [100]. In the present study, application of endophytic *P. aeruginosa* MF-30 promoted the lateral root formation and it was depicted from the enhanced accumulation of fresh and dry weight of root under pathogenic challenged conditions. It was obvious and well documented that well-established roots absorb high amounts of water and nutrients from the soil, which leads to better plant growth and development [38,39]. Results indicated that significantly higher shoot weight was recorded in the plants bio-primed with MF-30 and untreated healthy control (Table 2). The least biomass was reported in the plants treated with pathogen alone as compared to other treatments and untreated healthy control (T_6_). Studies clearly indicated that endophytic bacteria modulate biochemical pathways in the plants and elicited plant defense [101,102]. In the present study, bio-priming of the plants with *P. aeruginosa* MF-30 and subsequent foliar spray of culture suspension of MF-30 significantly activated defense enzymes in the plant and thus restricted the disease progression. However, maximum disease severity and lesion length were recorded in the plants inoculated with *R. solani* alone. Plants bio-primed with bioagents respond faster and show stronger activation of cellular defense responses after pathogen challenge compared to control plants. Significantly higher induction and accumulation of antioxidant and defense-related enzymes in the plants which are pretreated with biological agents interact in a cooperative manner and lead to better plant growth and health even in a stress condition. These results are also in agreement with the finding of earlier research [33,42,95]. The present study suggested that the seed bio-priming increases the potentiation of cellular defense responses with up-regulation of defense signaling cascades might be of great advantage for stressed plants.

## 5. Conclusions

The seed bio-priming with endophytic *P. aeruginosa* MF-30 and subsequent foliar spray of culture suspension of MF-30 resulted in significant enhancement of anti-oxidative defense enzymes in the maize plant with a simultaneous decrease in the H_2_O_2_ concentration. The modulated plant defense under bio-primed condition is further characterized by differential tissue-specific and temporal expression of pathogenesis-related gene (*zmPR-1* and *zmPR-10*) transcript, activation of phenylpropanoid pathway, and enhanced accumulation of proline. It was also observed that application of endophyte *P. aeruginosa* MF-30 significantly decreased the disease severity and lesion length with increased accumulation of plant root and shoot biomass in the maize plants pre-inoculated with *R. solani.* These findings add new and rather clear-cut information on microbes, more specifically endophytic *P. aeruginosa* MF-30-mediated mechanisms of induced systemic resistance in maize against *R. solani*.

## Figures and Tables

**Table 1:** Primers used for qRT-PCR in the gene expression study.

S. No.	Gene Name	Primers (5′–3′)
1.	*zmPR-1*	TCAGTCATGCCGTTCAGCTT
		TTGTCCGCGTCCAGGAA
2.	*zmPR-10*	CAACCCGGAAGCCTACAACTAG
		GAAATCCGTTCCCCATCGA
3.	*zmACTIN*	GGGATTGCCGATCGTATGAG
		GAGCCACCGATCCAGACACT

*zm—Zea mays*; *PR*—pathogenesis-related protein.

**Table 2:** Effect of seed biopriming with *P. aeruginosa* MF-30 on plant biomass accumulation in maize at 30 DAPI under nethouse conditions.

Treatments	Fresh wt. of Shoot (g)	Fresh wt. of Root (g)	Dry wt. of Shoot (g)	Dry wt. of Root (g)
T_1_	25.15 ± 1.05 ^f^	20.22 ± 0.45 ^e^	7.50 ± 0.15 ^e^	4.00 ± 0.05 ^f^
T_2_	32.42 ± 0.96 ^d^	24.66 ± 0.66 ^c^	8.54 ± 0.11 ^d^	5.05 ± 0.06 ^d^
T_3_	44.25 ± 1.00 ^b^	28.45 ± 1.05 ^b^	10.25 ± 0.15 ^b^	6.75 ± 0.10 ^b^
T_4_	30.25 ± 1.10 ^e^	22.75 ± 1.25 ^d^	8.02 ± 0.09 ^d^	4.78 ± 0.11 ^e^
T_5_	38.67 ± 0.75 ^c^	25.25 ± 0.85 ^c^	9.15 ± 0.07 ^c^	5.86 ± 0.05 ^c^
T_6_	50.46 ± 1.33 ^a^	30.40 ± 1.12 ^a^	12.03 ± 0.10 ^a^	7.20 ± 0.08 ^a^

where, T_1_-plants inoculated with *R. solani* alone; T_2_-*R. solani* + seed bio-primed with *P. aeruginosa* MF-30; T_3_-*R. solani* + seed bio-primed with *P. aeruginosa* MF-30 + foliar spray of MF-30; T_4_-*R. solani* + foliar spray of culture filtrate of MF-30; T_5_-*R. solani* + seed bio-primed with *P. aeruginosa* MF-30 + foliar spray of culture filtrate of MF-30; T_6_-Control (untreated). Data are mean ± SEM (*n* = 5), data with different letters show significant difference in column data in randomized block design test at *p* < 0.05 under Duncan’s multiple range test.

## Supplementary Materials

The following are available online at https://www.mdpi.com/1660-4601/17/4/1396/s1, Figure S1: GFP-tagging of *Pseudomonas aeruginosa* MF-30, Figure S2: Qualitative estimation of plant growth promoting and biocontrol attributes in *Pseudomonas aeruginosa* MF-30, Table S1: Qualitative estimation of plant growth promoting and biocontrol attributes in *Pseudomonas aeruginosa* MF-30.
