# Supplementary material for: Seed Biopriming with Microbial Inoculant Triggers Local and Systemic Defense Responses against Rhizoctonia solani Causing Banded Leaf and Sheath Blight in Maize (Zea mays L.)
Source: Int J Environ Res Public Health. 2020 Feb 21;17(4):1396. doi: 10.3390/ijerph17041396 (PMC7068308; doi:10.3390/ijerph17041396)
Supplement: Supplementary file 1 [file ijerph-17-01396-s001.pdf]

Supplementary Material

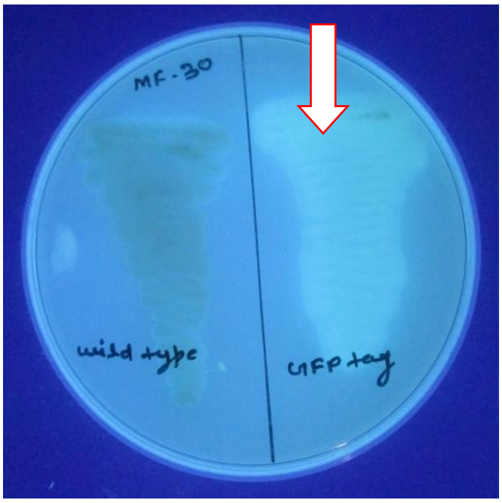

Figure S1. GFP-tagging of *Pseudomonas aeruginosa* MF-30.

Table S1. Qualitative estimation of plant growth promoting and biocontrol attributes in *Pseudomonas aeruginosa* MF-30.

S.No.	Plant growth promoting attributes	Presence/absence
1.	Phosphorus solubilization	+ve
2.	Potash solubilization	+ve
3.	Zinc solubilization	+ve
4.	IAA production	+ve
5.	HCN production	+ve
6.	Siderophore production	+ve
7.	Ammonia production	+ve
8.	Amylase activity	+ve
9.	Cellulase activity	+ve
10.	Pectinase activity	+ve
11.	Chitinase activity	+ve

S.No.	Plant growth promoting attributes	Presence/absence
1.	Phosphorus solubilization	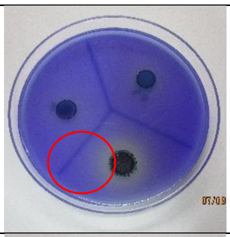
2.	Potash solubilization	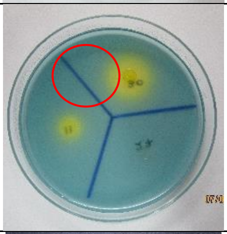
3.	Zinc solubilization	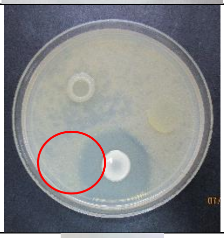
4.	IAA production	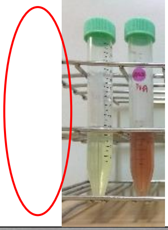
5.	HCN production	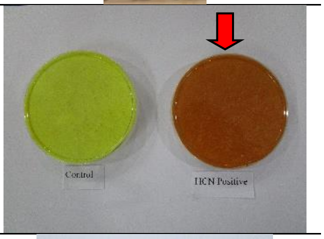
6.	Siderophore production	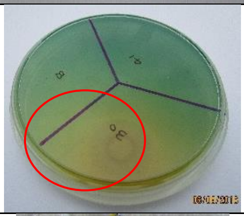
7.	Ammonia production	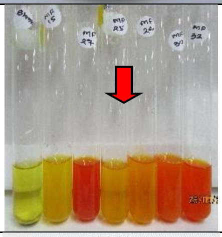
8.	Amylase activity	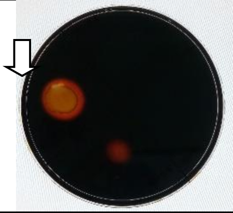

9.	Cellulase activity	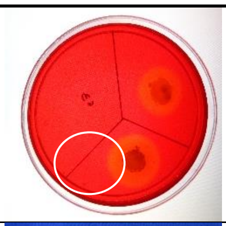
10.	Pectinase activity	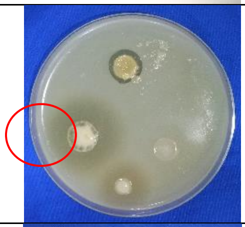
11.	Chitinase activity	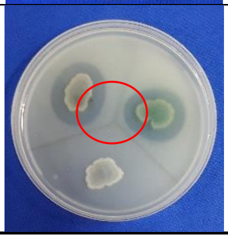

Figure S2. Qualitative estimation of plant growth promoting and biocontrol attributes in *Pseudomonas aeruginosa* MF-30.
